# Influenza

**Published:** 1916-04

**Authors:** 


**Influenza.** Anna R. Williams of the N. Y. Dept. of Health, in an examination of 50 cases of "influenza" obtained the following cultures: streptococcus 26, diplococcus lanceolatus 19, micrococcus catarrhalis 18, **Bacillus Influenzae 9**, staphylococcus pyogenes 9, bacillus capsulatus 6, bacillus bronchisepticus 2, Vincent's bacillus 1. (Note that the figures show joint infection in many cases, the associations not being statistically given). A few months ago, the influenza bacillus was found in only 1 case in 20 of grippe. (Boston M. & S. Jour., March 2.)

